# A conserved pulvinar projection to the amygdala revealed in macaque monkeys (*Macaca mulatta*)

**DOI:** 10.1007/s00429-026-03145-1

**Published:** 2026-06-30

**Authors:** Mary K. L. Baldwin, Arya Mohanty, Alexander C. Cummins, Elisabeth A. Murray

**Affiliations:** Laboratory of Neuropsychology, National Institute of Mental Health, National Institutes of Health, Building 49, Suite 1B80, 49 Convent Drive, Bethesda, MD 20892-4415 USA

**Keywords:** Primate, Emotion, Affect, Faces

## Abstract

Understanding the organization and function of thalamic pulvinar projections to the amygdala is of interest due to the proposal that this projection provides the amygdala with short-latency visual sensory input that eludes conscious awareness. However, most reports in primates have emphasized a projection from the multimodal medial pulvinar—a pulvinar division unique to primates—versus projections from visual pulvinar divisions (inferior or lateral). Further, reports in other closely related species such as tree shrews and rodents have yielded inconsistent results relative to primates when homology is considered. In these species, subdivisions of the lateral posterior/pulvinar complex, which are homologous to the visual inferior pulvinar of primates, project to the amygdala. Such a difference in pulvino-amygdala connections across these closely related species would be surprising. However, modern methods that reveal subdivisions of the pulvinar were lacking in previous anatomical studies of primate pulvino-amygdala connections. To better understand whether a major shift in pulvino-amygdala projections is truly present across species, we reevaluated the locations of pulvinar neurons projecting to the amygdala in rhesus monkeys (*Macaca mulatta*) using robust anatomical markers for delineating divisions of the pulvinar, and specifically highlighting the border between the medial and inferior pulvinar. Our findings show definitively that pulvino-amygdala projections in macaques share both conserved, via the visual inferior pulvinar, and novel, via the medial pulvinar, profiles. Further, our data provide a refinement in the available routes via which visual information could reach the amygdala, one that includes the inferior pulvinar nucleus of the thalamus.

## Introduction

A ‘fast pathway’ proceeding from the superior colliculus (SC) to the pulvinar nucleus of the thalamus to the amygdala has repeatedly been invoked to account for both rapid responses to visually detected threats and the ability of blindsight patients to detect and process stimuli in the ‘blind’ visual field (Morris et al. 1999; Morris et al. 2001; de Gelder et al. 1999; Vuilleumier et al. 2003; Lindell et al. 2005; 1999; Tamietto and de Gelder 2010; Pessoa and Adolphs 2010; Bertini et al. 2018; Soares et al. 2017: Mc Fadyen et al. 2019; Kragel et al. 2022). On this view, the SC-to-pulvinar-to-amygdala route would offer a proposed tri-synaptic pathway directly from the retina that could signal threats, one that bypasses the series of cortical fields in the ventral visual stream that are thought to process stimulus identity. To test the idea of a fast pathway in nonhuman primates, neurophysiologists have recorded from points along the putative SC-pulvinar-amygdala pathway while macaque monkeys viewed threatening stimuli. Lending support to the ‘fast pathway’ proposal, many neurons in the pulvinar and amygdala respond to images of snakes and faces (Maior et al. 2010; Le et al. 2013; 2014; Dinh et al. 2022), the two categories of stimuli most often used to evaluate responsiveness to threat in primates (Soares et al. 2017). Also consistent with the idea of a fast pathway to convey threat that bypasses cortical processing, Inagaki et al. (2023) showed that neuronal responses to faces occur faster in the macaque amygdala than within cortical fields within the visual inferior temporal cortex known to project to the amygdala.

**Fig. 1:**
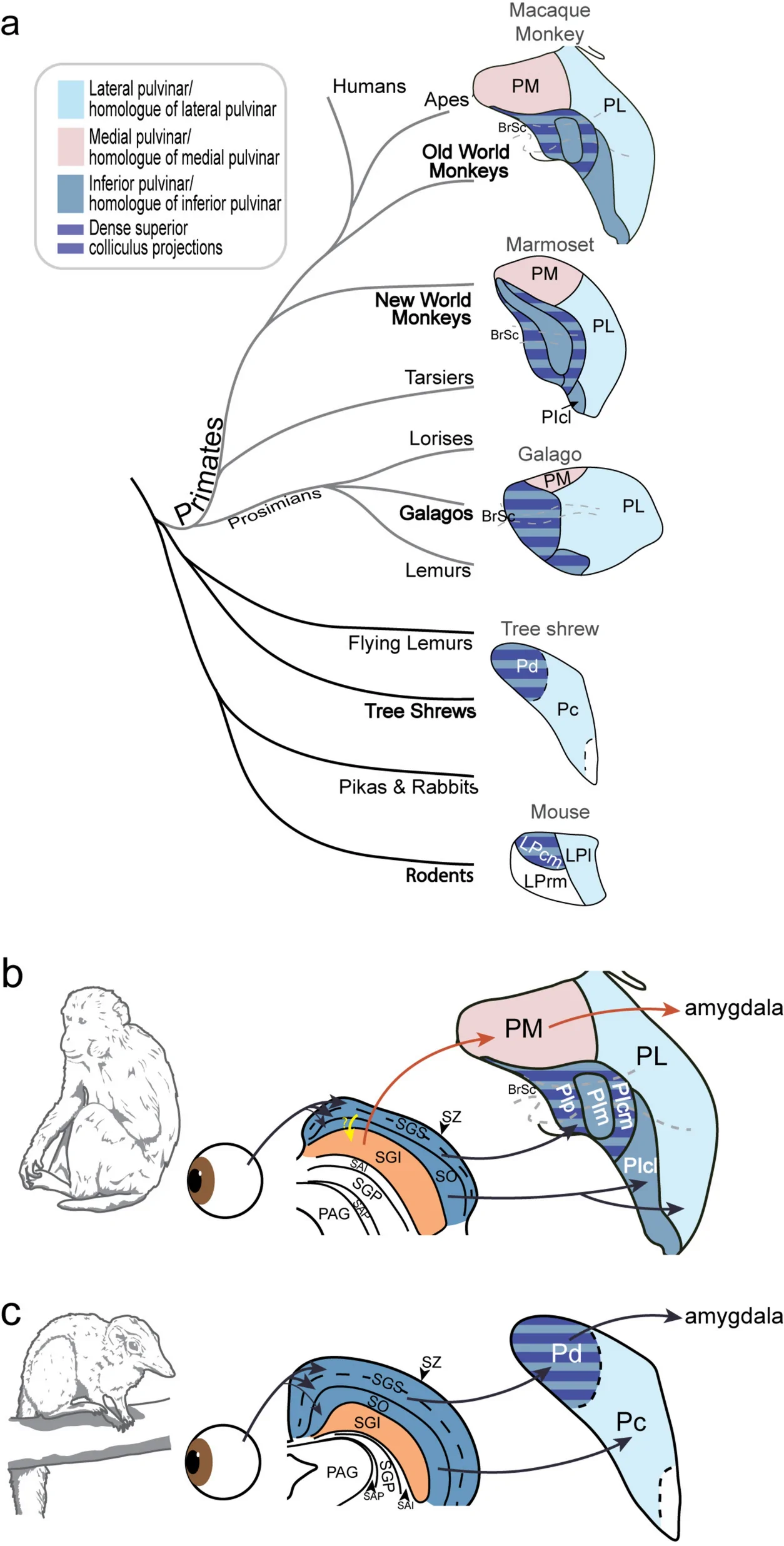
**A** Phylogenetic tree of Euarchontoglires, a clade that includes rodents, tree shrews, and primates. Each branching point indicates a point in time when currently living mammals shared a common ancestor. Time moves from left to right with the past to the left and current day to the far right. To the right of the phylogenetic tree are schematic coronal views of our current understanding of the organization of the pulvinar complex. Colors indicate widely accepted homologues. The visual pulvinar is composed of two main divisions: the lateral pulvinar (light blue) and the inferior pulvinar (dark blue). The medial pulvinar, which is multimodal, is shaded pink. This division of the pulvinar may be unique to primates; evidence for a similar structure in tree shrews and rodents has not been reported. Divisions of the pulvinar that receive dense SC input are shaded with dark blue stripes. **b** Summary of known projection patterns from the SC to the pulvinar complex in macaque monkeys, as well as projections from the pulvinar to the amygdala. Dashed gray lines show the location of the BrSC. **c**. Summary of known projection patterns from the SC to the pulvinar in tree shrews. Note that in tree shrews, it is Pd—the division of the pulvinar that receives direct and dense projections from the superficial layers of the SC—that gives rise to projections to the amygdala (Day-Brown 2010). In mice (refer to **a**), the LPcm projects to the amygdala (Wei et al. 2015). This division also receives inputs from the superficial layers of the SC. Abbreviations: BrSC, brachium of the superior colliculus; LPcm, caudomedial lateral posterior nucleus; LPl, lateral lateral posterior nucleus; LPrm, rostromedial lateral posterior nucleus; PAG, periaqueductal gray; Pc, central pulvinar; Pd, dorsal pulvinar; PI, inferior pulvinar; PIcl, central lateral inferior pulvinar; PIcm, central medial inferior pulvinar; PIm, medial inferior pulvinar; PIp, posterior inferior pulvinar; PL, lateral pulvinar; PM, medial pulvinar; SAI, stratum album intermedium; SAP, stratum album profundum; SGS, stratum griseum superficiale; SGP, stratum griseum profundum; SO, stratum opticum; SZ, stratum zonale

In macaques, most anatomical studies emphasize the medial pulvinar as the source of amygdala inputs from the pulvinar, and therefore the important link between the SC and amygdala for providing rapidly conveyed visual sensory information. However, this claim requires the resolution of a few major issues. The first is how the medial pulvinar is receiving visual information. Past reports have shown that it is the intermediate layers of the SC that project to the medial pulvinar (Benevento and Standage 1983) (Fig. 1a). The intermediate layers receive neither direct retinal input nor input from early visual cortical fields. In addition, neurons in the intermediate layers of the SC show responses to multimodal stimuli (Wallace and Stein 1996; Wallace et al. 1996). Instead, it is the superficial layers of the SC that receive direct retinal input (Hubel et al. 1975; Pollack and Hickey 1979; Dillbeck et al. 2022) and projections from early cortical visual areas including V1 and V2 (Lund et al. 1975; Fries et al. 1983; Fries 1984; Abel et al. 1997; Lock et al. 2003; Cerkevich et al. 2014). Further, cells in the superficial layers of the SC respond to visual stimuli (Marrocco and Li 1977; Moors and Vendrik 1979; Rizzolatti et al. 1980). Thus, if the SC were conveying important visual signals to the amygdala, the superficial layers would seem the most likely source of this information.

A second major issue is the apparent discrepancy in the origins of pulvinar projections to the amygdala when comparing primates to nonprimate species (Fig. 1a, b). In nonprimate species, divisions of the pulvinar/lateral posterior nucleus that are homologous to the inferior visual pulvinar in primates are known to give rise to projections to the amygdala. These divisions include the dorsal pulvinar (Pd) in tree shrews (Day-Brown et al. 2010) and the caudomedial lateral posterior nucleus (LPcm) in rodents (Wei et al. 2015; Doron and LeDoux 1999). Whereas one report has suggested that neurons in the inferior pulvinar or lateral pulvinar of macaques give rise to at least some projections to the amygdala (Elorette et al. 2018), this was based on a single case. Most anatomical studies report that the main input to the amygdala arises from neurons in the multisensory medial pulvinar (Jones and Burton 1976; Norita and Kawamura 1980; Rafal et al. 2015; Elorette et al. 2018). An additional study (Inagaki et al. 2023), using transneuronal transport of rabies virus following injections into the amygdala, likewise emphasizes projections from the medial pulvinar to the amygdala. Yet, retrogradely labeled cells were also observed within the inferior and lateral pulvinar. Because of the nature of the study, however, it is uncertain if the label in the inferior and lateral pulvinar is monosynaptic or due instead to multi-synaptic thalamo-cortico-amygdala connections as has been reported in mice (Zhou et al. 2018). Overall, the discrepant findings of what divisions of the pulvinar project to the amygdala in tree shrews and rodents compared to primates is curious as it implies a clear subcortical visual trajectory to the amygdala in nonprimate species, but a completely different input from a novel and multisensory division of the pulvinar in primates.

Most past studies in nonhuman primates relied on only Nissl-stained sections to delineate the pulvinar complex, and often used the brachium of the superior colliculus (BrSC) to define the boundary between medial and inferior/lateral pulvinar divisions, consistent with standard conventions in primate atlases (Olszewski 1952; Paxinos et al. 2000; Saleem and Logothetis 2012; 2007) available at the times of the studies. However, since the mid 1990’s it has become clear that the BrSC does not provide an appropriate border, especially pertaining to the border between the medial and inferior pulvinar. Specifically, in tissue stained for acetylcholine esterase (AChE) or calbindin, it is evident that parts of the inferior pulvinar, including the dorsal aspect of the posterior (PIp) and central medial (PIcm) inferior pulvinar, are present above the white matter tracts making up the BrSC (Gutierrez et al. 1995; Stepniewska and Kaas 1997). More recently, vesicular glutamate transporter 2 (VGLUT2) protein staining has also been found to be a robust marker for delineating borders of some inferior pulvinar divisions in primates and, like AChE staining, shows these divisions traversing the BrSC (Balaram et al. 2013; Baldwin and Krubitzer 2018). VGLUT2 immunohistochemical staining also reveals similar boundaries for the proposed homologous pulvinar subdivisions across rodents, tree shrews, and primates (for review see Baldwin et al. 2017; Zhou et al. 2017). Thus, the question of whether primates have a uniquely derived pathway of visual information to the amygdala that shares no homology with other closely related species could be resolved with better delineation of the primate pulvinar complex. To address this issue, we combined anatomical tract tracing with histological processing of the pulvinar to precisely identify the locations of amygdala-projecting cells within specific subdivisions of the pulvinar. We placed retrograde anatomical tracers within the lateral amygdala, then determined the location of labeled cells with respect to the boundaries of pulvinar subdivisions identified using stains for AChE and VGLUT2.

Our findings reveal that projections from the macaque pulvinar to the amygdala arise from both the medial division of the pulvinar as well as from subdivisions of the inferior pulvinar, mainly the posterior inferior pulvinar, PIp. We propose that the projections arising from the inferior pulvinar are those that could provide fast visual input to the amygdala; PIp is known to receive input from layers of the SC that receive both direct retinal input and input from early visual cortical areas. Additional studies are needed to elucidate the functions of the conserved (through the inferior pulvinar) vs. novel (through the medial pulvinar) projection types observed across taxa.

## Methods

### Subjects

**Fig. 2:**
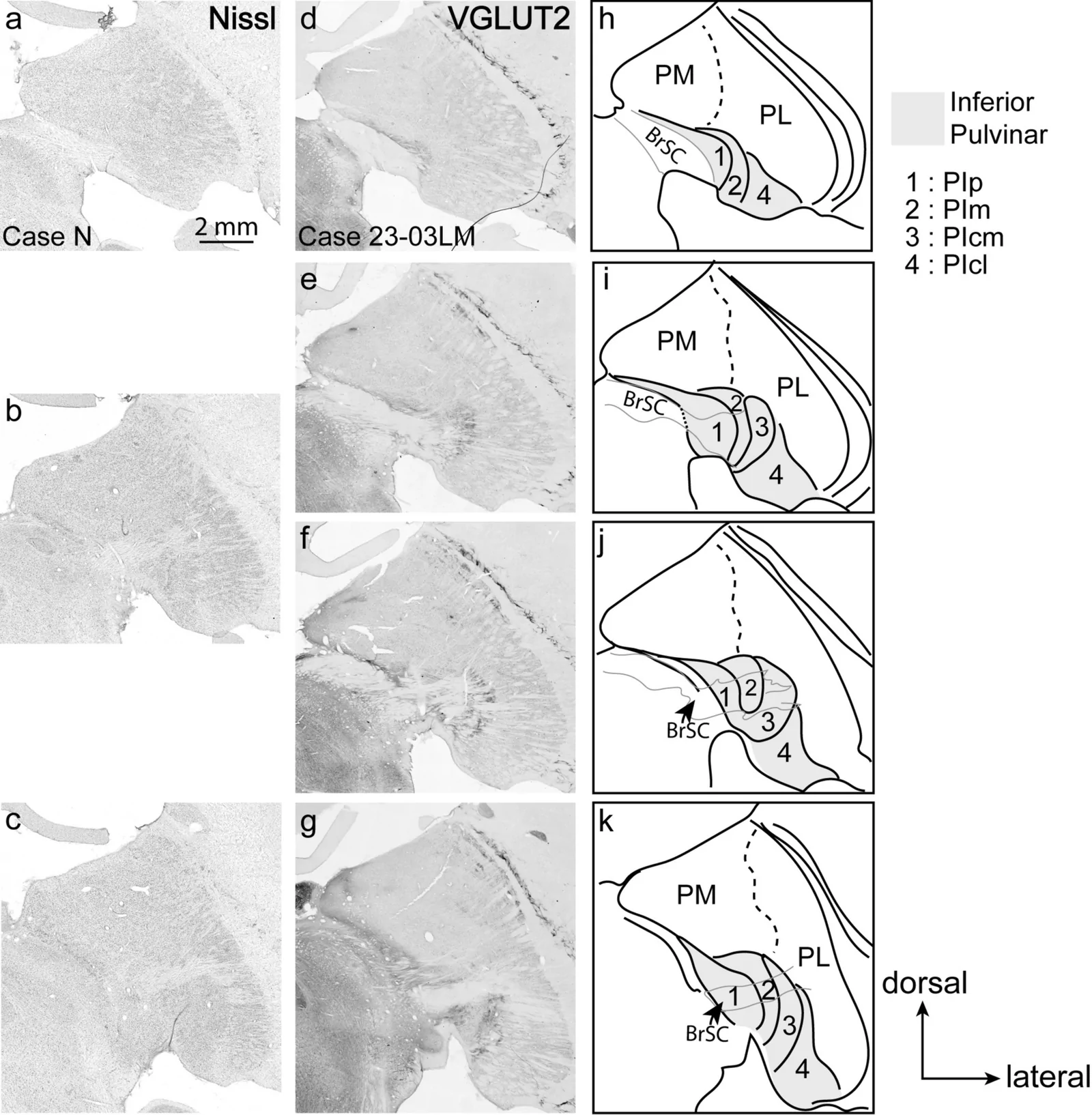
Anatomical borders of the pulvinar complex revealed using VGLUT2 staining. **a**, **b**, and **c**, show AP-matched sections of the pulvinar stained for Nissl substance. The medial pulvinar can be distinguished from the inferior and lateral pulvinar by less densely packed Nissl-stained cells. **d-g**. Photomicrographs of VGLUT2-stained sections through the pulvinar. Borders of specific divisions of the pulvinar are more robust in VGLUT2-stained tissue (panels **d**-**g**) relative to Nissl-stained tissue (**a-c**). PIp and PIcm both stain darkly for VGLUT2, while PIm and PIcl stain lightly for VGLUT2. Note that PIp and PIcm divisions of the inferior pulvinar as revealed with VGLUT2 progress dorsal to the white matter tracts of the BrSC. The medial pulvinar can be distinguished from the inferior pulvinar and the lateral pulvinar by having relatively little VGLUT2 staining. **h–k.** Schematic drawings showing the borders of the pulvinar as revealed in VGLUT2-stained sections. The inferior pulvinar divisions are shaded gray and the 4 main divisions are indicated using 1 (PIp), 2 (PIm), 3 (PIcm) and 4 (PIcl). The limitans nucleus also stains darkly for VGLUT2, and it seems to merge with PIp; however the limitans is present mostly along the anterior portion of the pulvinar and cells in this division stain more darkly, have an elongated morphology, and are more concentrated than the cells found in PIp in Nissl preparations. Images are taken from 2 different cases. Abbreviations: PM, medial pulvinar; PL, lateral pulvinar; PIp, posterior inferior pulvinar; PIm, middle inferior pulvinar; PIcm, central medial inferior pulvinar; PIcl, central lateral inferior pulvinar. More posterior sections through the pulvinar are located in the top panels while anterior sections progress downward with **k** representing the most anterior pulvinar section shown. The most posterior levels and rostral third of the pulvinar complex are not represented. Wavy gray lines in **h–k** indicate location of the BrSC

We studied 4 macaque monkeys (*Macaca mulatta*; 3 female, 1 male) ranging in age from 8 to 11 years and weighing between 9.1 and 14 kg. Three monkeys received bilateral injections of anatomical tracers, including Fluoro Ruby (FR), Fluoro Emerald (FE), lucifer yellow (LY) and cholera toxin subunit B (CTB), into the amygdala complex. The fourth animal was used to compare Nissl-stained tissue within the pulvinar to the AChE and VGLUT2 stained sections in the cases of this study (Fig. 2). All procedures were reviewed and approved by the National Institute of Mental Health Animal Care and Use Committee and followed the Guide for the Care and Use of Laboratory Animals.

### MR scans and surgical planning

One to three weeks prior to surgery, structural magnetic resonance imaging (MRI) scans were acquired using a 3 T Siemens PRISMA scanner. Monkeys were sedated with an initial dose of ketamine (10 mg/kg: IM) and Dexdomitor (0.01 mg/kg: IM), with supplemental IM doses (5 mg/kg ketamine, 0.01 mg/kg Dexdomitor) given every half hour or as needed. Animals were also given glycopyrrolate (0.015 mg/kg: IM) and ketoprofen (2 mg/kg: IM). Once anesthetized, monkeys were placed within an MRI-compatible stereotaxic frame. A tooth marker was used to record tooth position so that head placement in the scanner could be reproduced at the time of surgery. This was done to ensure that injection target locations based on the MRI scan would apply during surgery. The ear bars of the stereotaxic frame were filled with vitamin E to provide contrast in MR images, which served as fiducial marks during surgical planning. Heart rate, blood pressure, SPO_2_ and expired CO_2_ values were recorded throughout the procedure. Once scans were complete, animals were returned to their home cage and monitored during recovery from anesthesia.

OsiriX MD software (Pixmeo SARL, Bernex, Switzerland) was used to view MR images and measure the location of the lateral amygdala relative to the ear bars of the stereotaxic frame, the superior sagittal sinus, and other cortical surface landmarks such as the arcuate sulcus. These measurements were used to plan the placement of anatomical tracers.

### Surgical procedures

Standard aseptic surgical procedures were used. Monkeys were anesthetized with ketamine (10 mg/kg, IM) and dexdomitor (0.01 mg/kg, IM) and maintained with isoflurane gas (1–3%, to effect). Ketoprofen and glycopyrrolate were also administered prophylactically. Throughout surgery, we monitored the monkey’s vital signs including heart rate, respiration rate, body temperature, exhaled/inhaled CO_2_ and blood oxygen saturation. Once a steady level of anesthesia was attained an incision was made over the scalp, the skull above the injection sites was removed and a small incision along the dura was performed. Injections of a mixture of bidirectional FR ((0.4–0.6 μl: 10% of at 50:50 cocktail of 3,000 mw (Invitrogen Cat # D3308, Lot # 1,696,889) and 10,000 mw (Invitrogen D1817, Lot #1,722,352)) in sterile saline and FE ((0.4–0.6 μl: 10% of at 50:50 cocktail of 3,000 mw (Invitrogen Cat # 3306, Lot #1,722,355) and 10,000 mw (Invitrogen Cat #D1820, LOT# 1,654,903)), and CTB (0.5 μl 1% in sterile saline, List Labs: Catalog #104, Lot #s 10433A and 10434A1), were placed within the lateral amygdala at different locations (see Table 1) using a 7000 series Hamilton syringe with a 30-degree beveled tip. Injections of Lucifer yellow (Invitrogen Cat #L1177) were also placed in two cases; this tracer failed to result in transport and is therefore not considered further. Injections of tracers were carried out over a period of 5 min and the syringes were left in place for 7–10 min after injection completion to prevent tracer leaking dorsally along the syringe needle track. Once tracers were placed, the dura was sutured, the bone flap was replaced and sutured in place, and the scalp was sutured closed. Animals were then given prophylactic antibiotics and analgesics, and were monitored postoperatively until fully recovered.

**Table 1:** Case summary

Case #	Tracer injected	Total volume	Nuclei and structures involved in the injection site	Pulvinar nuclei where labeled cells were observed	Figure
* N (anatomical Case) Right hemisphere	Anatomy	n/a	n/a	Pulvinar anatomy (Nissl)	2
23–02 Left hemisphere	FR	0.5 ul	Bi	PM and PIcl	5
	CTB	0.4 ul	Lateral Bi and Bm and dorsomedial LA and some white matter lateral to Ce	PM, PIp, PIcl	5
	LY	0.5 ul	LA	Failed transport	n/a
23–03 Right hemisphere	CTB	0.5 ul	dorsolateral LA	PM, PIp, PIcl, PL	3
	FR	0.5 ul	White matter	PM	3
23–03 Left hemisphere	Anatomy	n/a	n/a	Pulvinar anatomy (VGLUT2)	2
	LY	0.5 ul	LA	Failed transport	n/a
	FR	0.5 ul	laterodorsal LA	PM and PIp	4
24–07 Right hemisphere	FR	0.65 ul	Bm and dorsomedial LA	PM, PIp	6
	FE	0.6 ul	Bm and Bi	PM	6
24–07 Left hemisphere	CTB	0.6 ul	Failed injection	n/a	n/a
	FR	0.6 ul	Dorsoanterior LA, dorsal Basal and white matter dorsal and lateral to LA	PM, PIp, PIcl, PL	7

*N is an archival case that was added specifically to compare Nissl staining to the VGLUT2 and AChE staining. This case did not receive anatomical tracer injections

Seven to eleven days after tracer injections, animals were sedated with an injection of ketamine (10 mg/kg IM) and then sacrificed with an overdose of Euthasol® and perfused transcardially with saline followed by 4% paraformaldehyde in 0.01 M PBS followed by 4% paraformaldehyde in 0.01 M PBS with 10% sucrose. The brain was removed, cut at the midline to separate the left and right hemispheres and placed in 30% sucrose solution for cryoprotection for 72 h prior to cutting. For the fourth case, which was used for cytoarchitectonic analysis, the monkey underwent a similar perfusion procedure with transcardial perfusion using saline followed by 4% paraformaldehyde. The brain was then cryoprotected with increasing concentrations of glycerols (Rosene et al. 1986).

All brains were then cut in the coronal plane at a thickness of 40 μm using a freezing microtome. For the tracer injection cases, sections were saved in a series of 6. One series was mounted directly onto slides for fluorescent analysis, another series was processed for AChE (Geneser-Jensen and Blackstad 1971) and two other series underwent immunohistological staining procedures for CTB tracer and vesicular glutamate transporter 2 (VGLUT2) (see Baldwin and Krubitzer 2018). The brain used for Nissl staining was saved in a series of 10, and one series was stained for thionin and another series was stained for AChE.

The protocols used for CTB and VGLUT2 immunohistological staining are outlined in previously published studies (Baldwin et al. 2011; Baldwin and Krubitzer 2018 etc.). Briefly, sections were rinsed in 0.1 M PBS 3 times for 5 min on a rocker at room temperature. Sections were then placed in a 0.01% hydrogen peroxidase solution in 0.01 M PBS for 10 min to quench endogenous peroxidase activity, then rinsed 3 times in 0.01 M PBS. Next, sections were incubated in 5% normal goat (VGLUT2) or horse (CTB) blocker with 0.05% triton X-100 for 2 h at room temperature. After this, sections were transferred to a 5% normal goat or horse blocker with the primary antibody (1:5000 for CTB, List Laboratories Inc catalog #703 and 1:2000 for VGLUT2, Millipore MAB554: See antibody Table 2) and placed on a rocker at 4 °C for 24 to 48 h. Next, the sections were rinsed 3 times for 5 min in 0.01 M PBS then placed in a 5% normal goat or horse serum blocker with 0.05% triton X-100 in 0.01 M PBS at room temperature for 90 min to 2 h. Sections were then rinsed 3 times for 5 min in 0.1 M PBS, then incubated in an avidin/biotin–peroxidase complex solution (ABC vector labs) in 0.01 M PBS overnight on a rocker at 4 °C. Finally, sections were rinsed 3 times in 0.01 M PBS and reacted in 0.1% 3′3’ diaminobenzidine, 0.001% hydrogen peroxidase, and 0.02% nickel ammonium sulfate solution in 0.01 M PB.

**Table 2:** Number and distribution of retrogradely labeled cells across pulvinar samples and cases

Case	Tracer	Pulvinar division	Section 1	Section 2	Section 3	Total	Percent total (%)
			# of labeled cells	# of labeled cells	# of labeled cells		
23–02 LH	CTB	PM	10	6	1	**17**	40.5
		PIp	7	5	11	**23**	54.8
		PIm	0	0	1	**1**	2.4
		PIcm	0	0	0	**0**	0
		PIcl	1	0	0	**1**	2.4
		PL	0	0	0	**0**	0
23–02 LH	FR	PM	3	5	0	**8**	88.9
		PIp	0	0	0	**0**	0
		PIm	0	0	0	**0**	0
		PIcm	0	0	0	**0**	0
		PIcl	1	0	0	**1**	11.1
		PL	0	0	0	**0**	0
23–03 RH	CTB	PM	42	36	16	**94**	52.8
		PIp	28	24	13	**65**	36.5
		PIm	0	0	0	**0**	0
		PIcm	0	2	1	**3**	1.7
		PIcl	8	5	1	**14**	7.9
		PL	1	1	0	**2**	1.1
23–03 RH	FR	PM	1	1	2	**4**	80
		PIp	0	0	1	**2**	20
		PIm	0	0	0	**0**	0
		PIcm	0	0	0	**0**	0
		PIcl	0	0	0	**0**	0
		PL	0	0	0	**0**	0
23–03 LH	FR	PM	3	2	5	**10**	66.7
		PIp	1	2	1	**4**	26.7
		PIm	0	0	0	**0**	0
		PIcm	0	0	0	**0**	0
		PIcl	0	0	1	**1**	6.7
		PL	0	0	0	**0**	0
24–07 RH	FR	PM	3	9	6	**18**	58.1
		PIp	2	3	4	**9**	29.0
		PIm	0	0	0	**0**	0
		PIcm	0	0	0	**0**	0
		PIcl	0	1	0	**1**	3.2
		PL	0	1	2	**3**	9.7
24–07 RH	FE	PM	1	0	0	**1**	50
		PIp	0	1	0	**1**	50
		PIm	0	0	0	**0**	0
		PIcm	0	0	0	**0**	0
		PIcl	0	0	0	**0**	0
		PL	0	0	0	**0**	0
24–07 LH	FR	PM	10	9	7	**26**	59.1
		PIp	6	1	6	**13**	29.5
		PIm	0	1	0	**1**	2.3
		PIcm	0	0	0	**0**	0
		PIcl	1	1	1	**3**	6.8
		PL	1	0	0	**1**	2.3

### Anatomical reconstructions

Labeled cells, tissue outlines, and blood vessels were plotted using a Neurolucida (MBF Bioscience, Williston, Vermont) XY stage encoding system attached to a Zeiss axioscope. Plots were then exported into Adobe Illustrator where they were aligned to images of adjacent tissue sections stained for VGLUT2 and AChE using common landmarks and blood vessels. After alignment of plots to anatomical borders, cell counts were obtained within each individual pulvinar nucleus using the document information window in Adobe Illustrator. Images of injection sites and histologically processed tissue sections were taken using an Olympus VS200 slide scanner. Images were adjusted for brightness and contrast and in some cases converted to black and white images in Adobe Photoshop but were otherwise unaltered.

## Results

Twelve tracer injections were placed within or near the amygdalae of 3 macaque monkeys. Of these, 8 injection sites resulted in the retrograde labeling of cells within the pulvinar complex: 2 sites were confined almost exclusively to the lateral nucleus, 2 were predominantly in the basal nucleus, 3 involved substantial portions of both the lateral and basal nuclei, and 1 site was located in the white matter just lateral to the amygdala (see Table 1). Though we attempted to contain our injection sites within the amygdala, some tracer did leak or contaminate tissue just dorsal to the amygdala. We disclose those instances in the provided photographs and reconstructions of each individual injection site.

### Anatomical borders

**Fig. 3:**
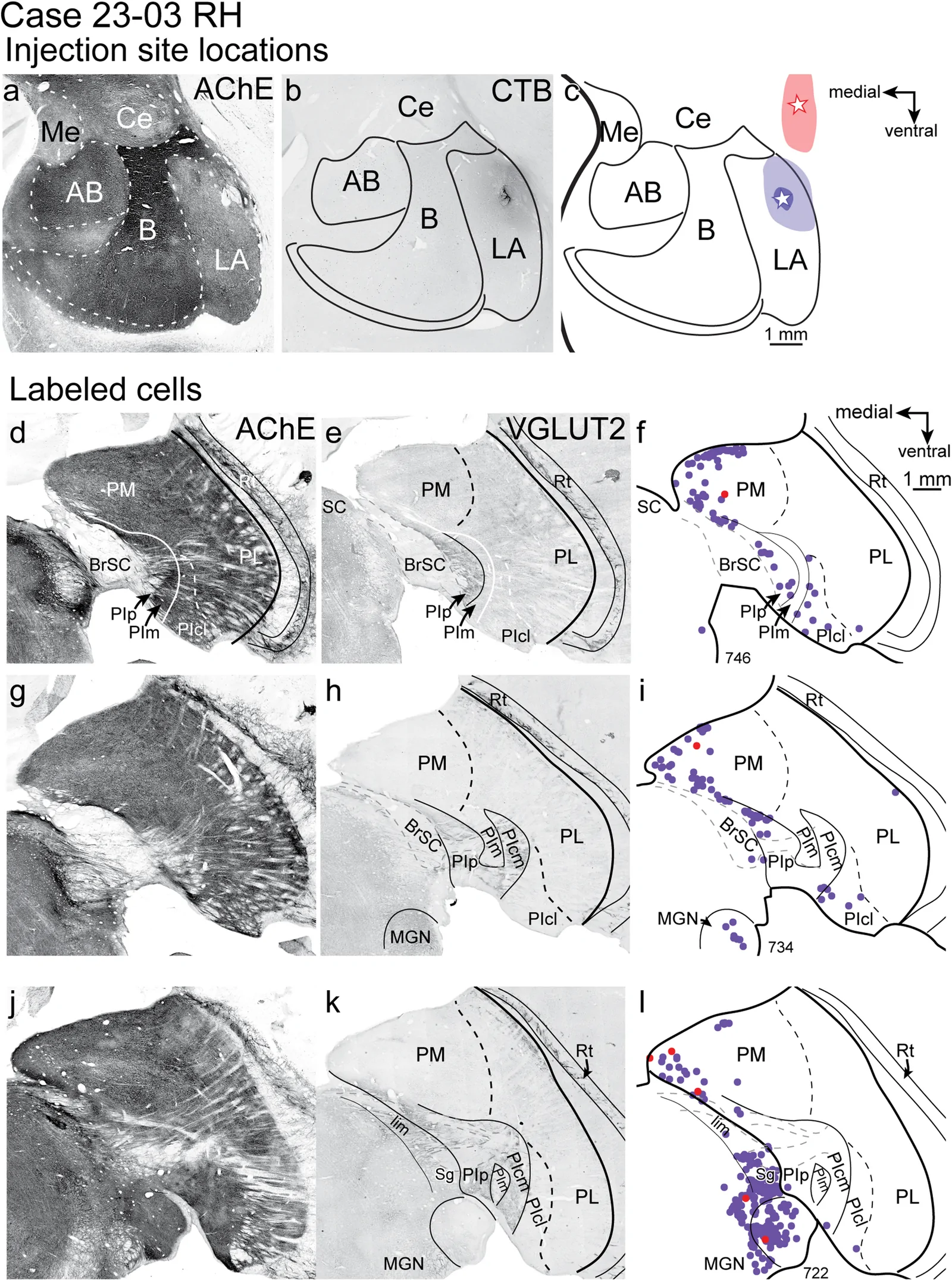
Reconstruction of labeled cells after a tracer injection into the lateral amygdala in case 23–03 RH. **a, b, c.** The top row of images shows the location of the CTB injection site (**b**) relative to anatomically defined borders based on the adjacent AChE (**a**) stained section. **c.** Reconstruction created by aligning **a** and **b**. The star indicates the CTB injection site core, while the purple shading represents the extent of tracer spread. Also shown is the location of the FR injection site and tracer spread (star and red/pink shading) from an adjacent section that was mounted and coverslipped for fluorescence analysis (not shown). **d-l.** Panels show the reconstruction of the location of labeled cells relative to anatomically defined borders based on AChE (**d, g, j**), and VGLUT2 (**e, h, k**) stained sections. Purple dots represent cells retrogradely labeled from the CTB injection, and red dots represent cells retrogradely labeled from the FR injection. Abbreviations and conventions as in Fig. 2. Posterior pulvinar sections are located at the top (**d-f**) with more anterior sections progressing down with the most anterior section shown in panel **l**. Dashed gray lines indicate location of the BrSC. Abbreviations: AB, accessory basal amygdala nucleus; AChE, acetylcholine esterase; B; Basal amygdala nucleus; BrSC, brachium of the superior colliculus; Ce, central amygdala nucleus; CTB, cholera toxin subunit B; LA, lateral amygdala nucleus; lim, limitans nucleus; Me, medial amygdala nucleus; MGN, medial geniculate nucleus; PM, medial pulvinar; PL, lateral pulvinar; PIp, posterior inferior pulvinar; PIm, middle inferior pulvinar; PIcm, central medial inferior pulvinar; PIcl, central lateral inferior pulvinar; Rt, reticular nucleus; Sg, suprageniculate nucleus; VGLUT2, vesicular glutamate transporter type 2

Borders of the amygdala nuclei and pulvinar subdivisions were determined using AChE and VGLUT2 histological staining. In many studies Nissl-stained sections have been used to determine the anatomical borders within the pulvinar, but in our experience borders for the divisions of the inferior pulvinar were much more robust and reliable using VGLUT2 staining (see Fig. 2 for comparison). Therefore, borders within the pulvinar were determined with a combination of AChE- and VGLUT2-stained sections (Figs. 2d-g, 3 and 5). The medial pulvinar can be distinguished from the inferior and lateral pulvinar by its lighter staining for VGLUT2 (Balaram et al. 2013) (Figs. 2, 3 and 5); the medial pulvinar also has a smoother appearance relative to the lateral pulvinar in AChE-stained sections (Figs. 3 and 5). Within the posterior aspect of the pulvinar, the medial pulvinar stains less darkly for AChE than the lateral and inferior pulvinar, but in more anterior sections the staining becomes darker making it more difficult to distinguish from surrounding pulvinar divisions.

The inferior pulvinar of macaques consists of 4 nuclei including PIp, PIm, PIcm, and PIcl. The staining properties of these subdivisions in macaques and New World monkeys have been well documented for AChE (Lysakowski et al. 1986; Gutierrez et al. 1995; Stepniewska et al. 1997; Gray et al. 1999) and VGLUT2 (Balaram et al. 2013; Baldwin and Krubitzer 2018). PIp and PIm stain darkly for AChE relative to surrounding structures including the medial pulvinar and PIcm, especially in posterior sections, while PIp and PIcm both stain darkly for VGLUT2 and PIm has little VGLUT2 staining (Figs. 2, 3 and 5). These staining properties are consistent across studied New and Old World monkeys (see Baldwin et al. 2017 and Baldwin and Krubitzer 2018 for reviews), and the dark VGLUT2 staining of some inferior pulvinar divisions in macaques and other monkeys matches the dark VGLUT2 staining in the proposed homologue of the inferior pulvinar in tree shrews and rodents (see Baldwin et al. 2017 for review).

Unlike the borders depicted in commonly used macaque atlases (Olszewski 1952; Paxinos et al. 2000; Saleem and Logothetis 2007, 2012), we found that parts of the inferior pulvinar, specifically, PIp, were present along the medial aspect of the pulvinar within the most posterior sections (Fig. 2d, 3e, 5h and k). Progressing anteriorly, other inferior pulvinar divisions emerge including PIm and PIcm and then eventually PIcl (Figs. 2 e–g, 3 h and k, 5n). Notably, portions of the inferior pulvinar are evident above the BrSC, a bundle of white matter tracts that bisects the pulvinar. As indicated in the Introduction, although for decades the BrSC was used to designate the border between the medial pulvinar and inferior pulvinar (Olszewski 1952; Walker 1938), anatomical studies by Gutierrez et al. (1995) and Stepniewska and Kaas (1997) provided evidence that parts of the inferior pulvinar extend slightly above the BrSC. This was evident in our own VGLUT2 staining: portions of PIp, PIm and PIcm extend above the BrSC (Fig. 2e, f, g). Finally, PIcl can be distinguished from the lateral pulvinar by having slightly less granular VGLUT2 staining (Fig. 2).

The borders of the amygdala are most easily delineated using AChE-stained sections (see Figs. 3a, 5a, d and 6a, d, 7a). For our purposes, determining the border between the lateral and basal nuclei of the amygdala was most important, and this border is clearly visible with AChE staining. The basal nucleus of the amygdala stains darker than the lateral nucleus (Girgis 1980; Amaral and Bessett, 1989; also see Figs. 3a, 5a and d, 6a and d, 7a).

### Connections

Case 23–03 RH contained a clean CTB injection site located within the dorsal lateral portion of the right lateral amygdala (Fig. 3a). There was no observable tracer contamination of the white matter tracks running lateral to the lateral nucleus nor involvement of the basal nucleus of the amygdala. Accordingly, this injection served as our most informative case for localizing neurons in the pulvinar that give rise to projections to the lateral nucleus. Labeled cells resulting from the CTB injection in case 23–03 RH were predominantly found within the dorsal and medial aspects of the medial pulvinar (PM); more central locations within this nucleus were void of labeled cells (Fig. 3i and l), and few to no labeled cells were located within the lateral aspect of the medial pulvinar close to the border with the lateral pulvinar. PIp also contained many labeled cells throughout its full dorsoventral extent caudally (Fig. 3f); only a few cells were located at more dorsal locations in more rostral sections (Fig. 3h). Labeled cells were also evident throughout the caudal aspect of PIcl (Fig. 3f, i and l). Finally, a few labeled cells were also evident in the lateral pulvinar.

Outside of the pulvinar labeled cells were found in the limitans nucleus, the suprageniculate nucleus, and the medial geniculate nucleus. These observations are consistent with past reports in both primates and rodents (LeDoux et al. 1984; Le Doux et al. 1990; Romanski and LeDoux 1992; Doron and LeDoux 1999; Linke et al. 1999; Keifer et al. 2015; Rafal and Koller 2025).

A FR injection site for this same case and hemisphere was located within the white matter just lateral and dorsal to the lateral nucleus (Fig. 3c). Though there was minimal label within the pulvinar from this injection site, some labeled cells were observed within PM and the medial geniculate nucleus; few were present in other locations. These labeled cells are most likely due to tracer uptake from axons of passage through this portion of the white matter.

**Fig. 4:**
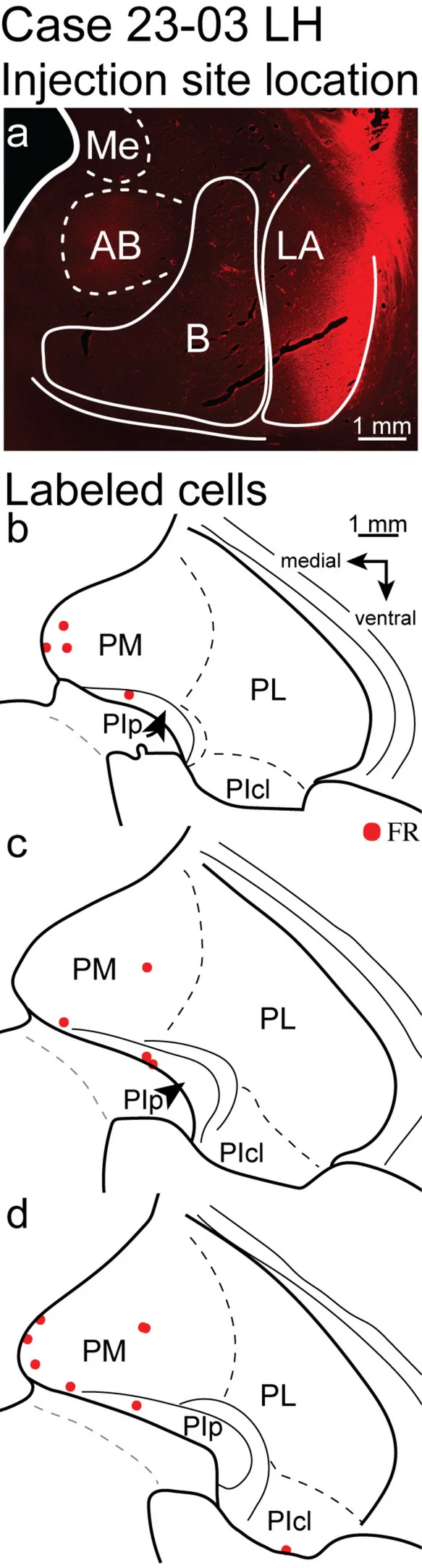
Reconstruction of labeled cells after an injection into the lateral amygdala and immediately adjacent white matter in case 23–03 LH. **a**. Photomicrograph of a FR injection site. The injection site was spread across the lateral amygdala and involved white matter lateral and dorsal to the amygdala. Only a few retrogradely labeled cells were observed; these were found within the medial pulvinar and some divisions of the inferior pulvinar (PIp and PIcl). Abbreviations and conventions as in Fig. 3

Case 23–03 LH contained an FR injection within the anterior portion of the lateral nucleus of the amygdala that extended into the immediately adjacent white matter tracts (Fig. 4a). Only a few retrogradely labeled cells were observed within the pulvinar; labeled cells were located primarily within PM, PIp and PIcl (Fig. 4b-d). Thus, our results for case 23–03 LH were consistent with the clean injection site within the lateral nucleus in case 23–03 RH.

**Fig. 5:**
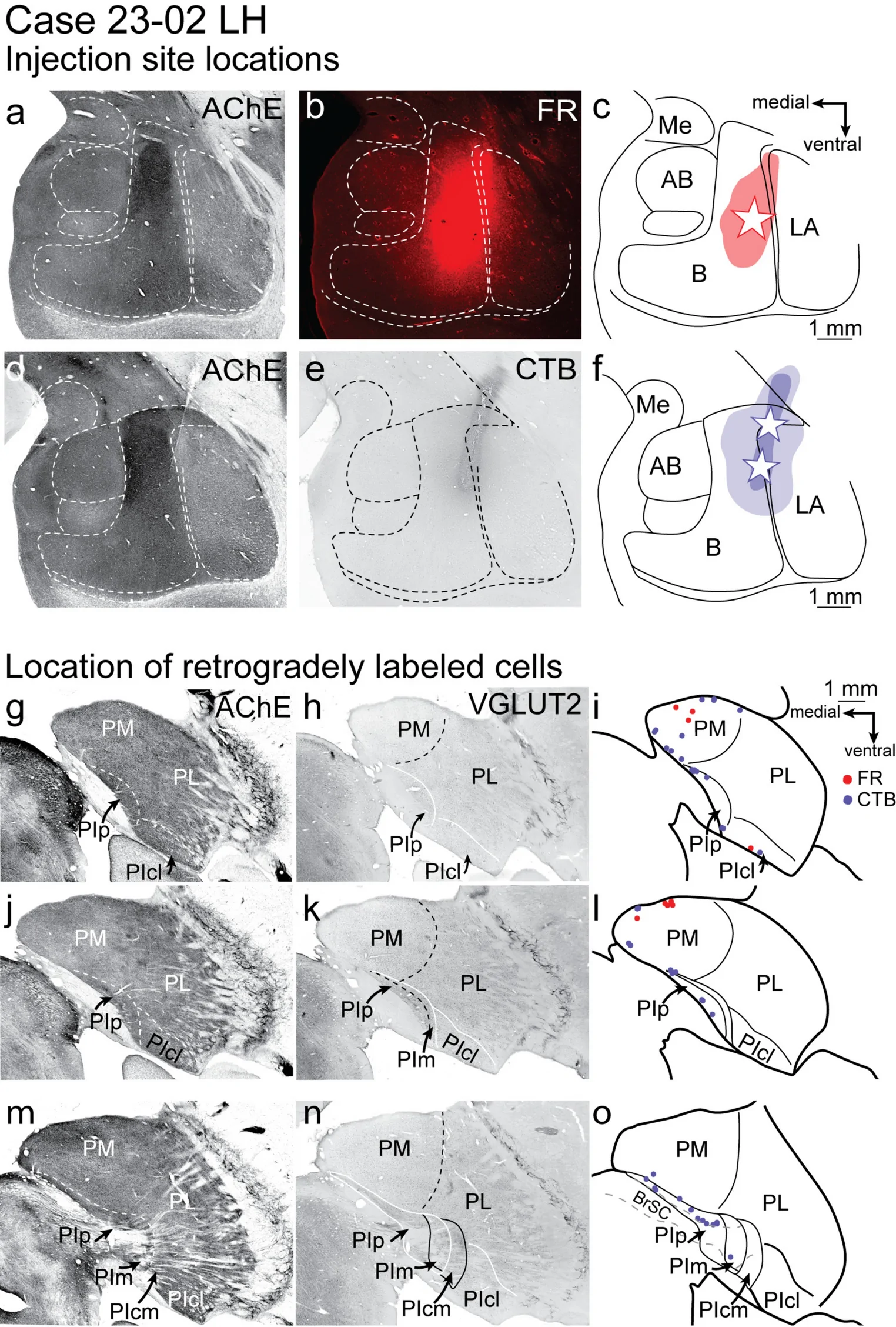
Reconstruction of the location of labeled cells after tracer injections into the lateral amygdala in case 23–02 LH. **a-c**. Location of FR injection. **d-f**. Location of CTB injection. **g-o**. Reconstructions of the locations of retrogradely labeled cells following the injections shown in **c** and **f**. Abbreviations and conventions as in Fig. 3

Two cases each had two injection sites involving the basal nucleus of the amygdala. The first was case 23–02 LH, in which an FR injection site was centrally located within the intermediate division of the basal nucleus. There was minimal to no tracer spread into the lateral nucleus (Fig. 5a-c). Retrogradely labeled FR cells were primarily located within PM with only one cell labeled within PIcl. No cells were observed within PIp from this injection site. This is in contrast to the CTB injection site for this same case (Fig. 5d-f), which involved both the basal nucleus as well as the lateral nucleus. Here, CTB labeled cells were located within PM, PIp and PIcl.

**Fig. 6:**
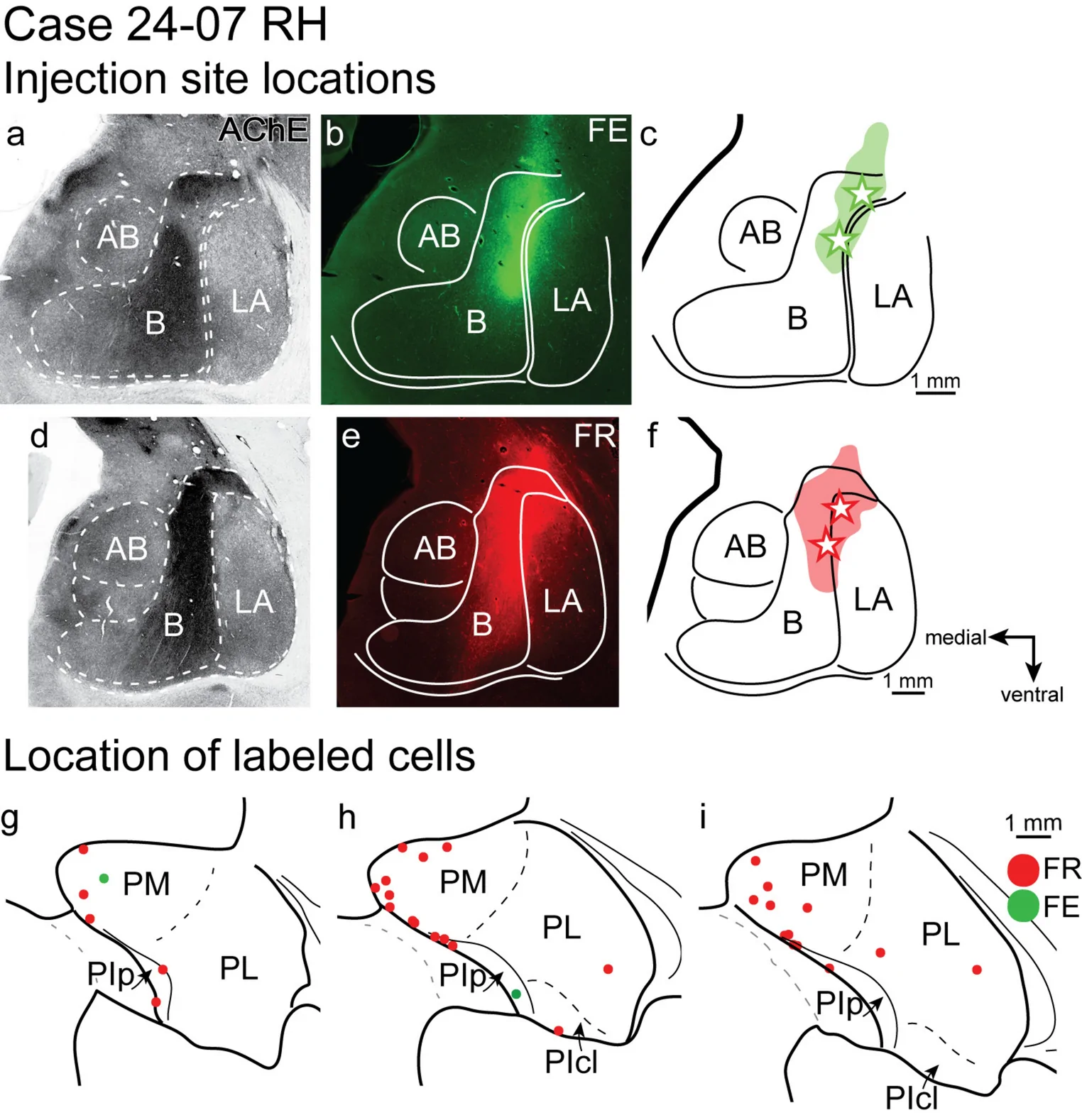
Distribution of labeled cells after tracer injections into the amygdala in case 24–07 RH. **a-c**. Location of FE injection. **d-f**. Location of FR injection. **g-i.** Locations of retrogradely labeled cells within the pulvinar resulting from injections shown in **c** and **f**. More posterior sections of the pulvinar are presented to the left (**g**) with more anterior sections progressing to the right (**h** then **i**). Abbreviations and conventions as in Fig. 3

The second case with tracer injections involving the basal nucleus was case 24–07 RH. This case had two injection sites that included the intermediate and magnocellular divisions of the basal nucleus (Fig. 6a-f); each injection site also involved small portions of the dorsomedial aspect of the lateral nucleus of the amygdala. The FE injection site (green) did not yield many labeled cells overall. However, retrogradely labeled cells within the pulvinar were observed in PM and PIp (Fig. 6g, h). The FR injection site resulted in labeled cells within the medial and dorsal aspects of PM as well as a small number of cells within PIp, PIcl and PL (Fig. 6g-i). Thus, injections involving the basal nucleus of the amygdala with minimal spread into the lateral nucleus (e.g., the FR injection in case 23–02 LH) receive proportionately more input from the medial pulvinar and only weak, if any, input from the inferior pulvinar. Indeed, it is possible that pulvino-amygdala projections arising from the inferior pulvinar only terminate in the lateral nucleus of the amygdala.

**Fig. 7:**
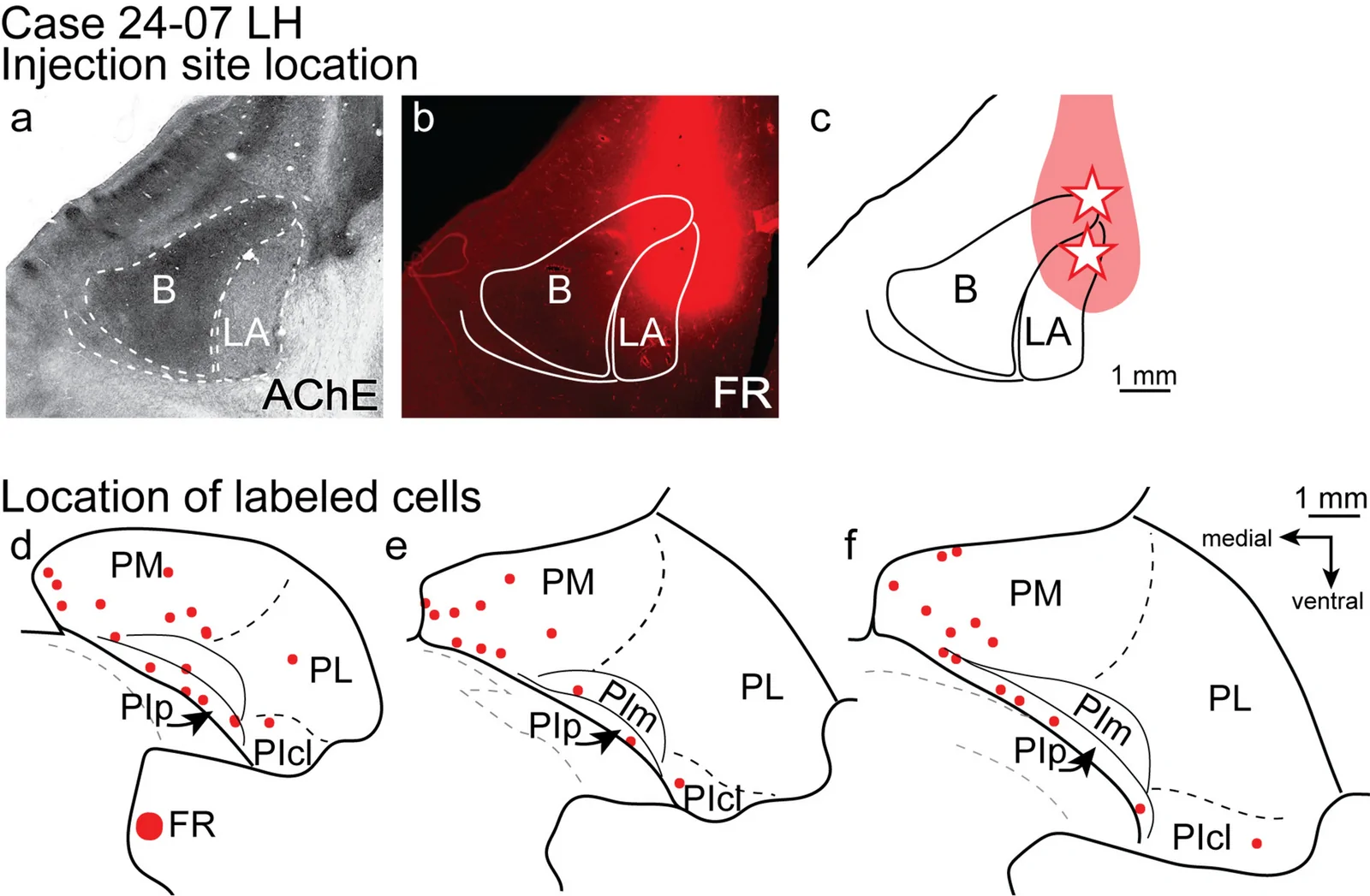
Reconstruction of labeled cells after a tracer injection in the anterior amygdala in case 24–07 LH. **a-c**. Location of injection of FR; the injection site included both the basal and lateral nuclei of the amygdala as well as white matter dorsally. **d-f**. Locations of retrogradely labeled cells, which were present within the inferior pulvinar (PIp and PIcl) as well as the medial pulvinar. Abbreviations and conventions as in Fig. 3

Finally, the FR injection site in case 24–07 LH included the anterior portions of both the left lateral and basal nuclei (Fig. 7a-c). This injection site resulted in retrogradely labeled cells primarily within PM, PIp, and PIcl; a few additional labeled cells were found in the lateral pulvinar at the most posterior extent (Fig. 7d).

### Cell counts

Our tracer study was not designed to be quantitative. Numbers of labeled cells can be influenced by many factors that we did not control for: size of injection, type of tracer and its efficacy of uptake by terminals, and more. Although cell counts need to be interpreted with caution, they may offer insight into the pattern of results. Table 2 provides a summary of the location and number of retrogradely labeled cells within the pulvinar for each case and injection site.

**Fig. 8:**
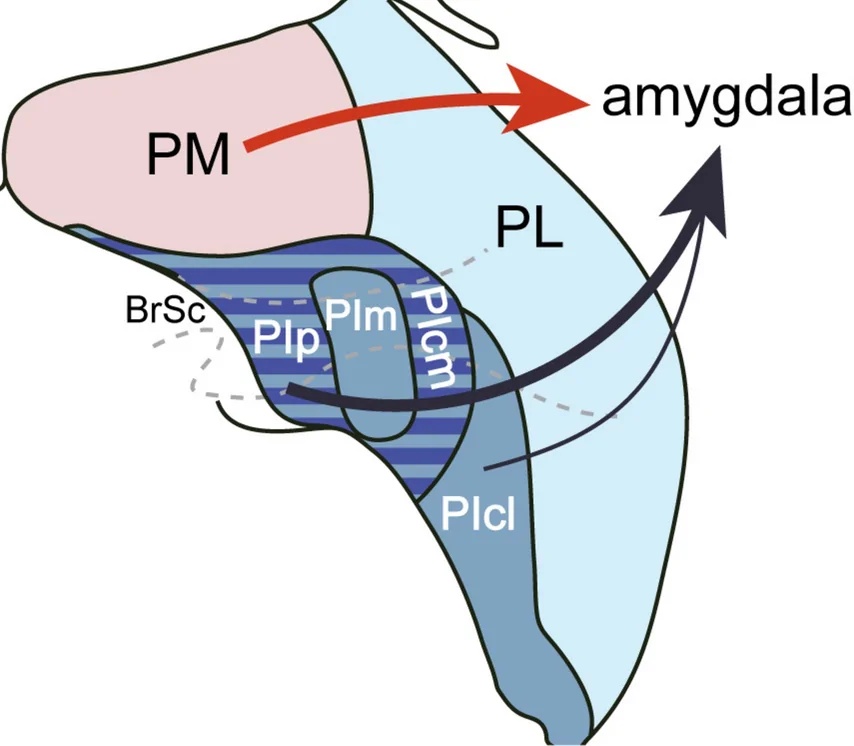
Summary of projections to the amygdala from the pulvinar complex in macaque monkeys. The cartoon depicts an iconic coronal view of the macaque pulvinar complex highlighting the known pulvinar subdivisions. The current study shows that there are strong projections from both the medial and inferior pulvinar (especially from PIp) to the amygdala in macaque monkeys. Abbreviations and shading conventions as in Fig. 1

Labeled cells were most prevalent within PM and PIp divisions of the pulvinar. Far fewer cells were observed within PIm, PIcm, PIcl, and PL. Overall, more labeled cells were observed within PM relative to PIp. For cases with injection sites within the amygdala, PM accounted for 40.6 to 88.9% of all labeled cells in the pulvinar (Table 2). By contrast, PIp accounted for 0% to 54.8% of all labeled cells (Table 2). In one case with few overall labeled cells (i.e. the FR injection site for case 23–02 LH; Fig. 5) and with an injection site centered within the basal amygdala nucleus, no (0%) labeled cells were observed within PIp and 88.9% of the labeled cells were observed within PM. In cases with CTB injections that involved the lateral nucleus of the amygdala (Figs. 3, 5), retrogradely labeled cells were found in roughly equal proportions within PM and PIp (PM, 40.5 to 52.8%; PIp, 36.5 to 54.8%) (Fig. 8).

In summary, injection sites that included the lateral nucleus of the amygdala consistently resulted in retrogradely labeled cells within the medial pulvinar and the inferior pulvinar including PIp, and PIcl, with a few labeled cells also found within the lateral pulvinar and PIm. Injection sites that included the basal nucleus of the amygdala resulted in proportionately more labeled cells within the medial pulvinar relative to the inferior pulvinar. The greater the extent of the lateral nucleus involved in the injection site, the greater the number of labeled cells within the inferior pulvinar subdivision PIp (Fig. 5). Thus, the inferior pulvinar seems to project predominantly to the lateral nucleus of the amygdala, whereas the medial pulvinar appears to project to both the lateral and basal nuclei of the amygdala.

## Discussion

The aim of the present study is to assess the precise origins of cells within the pulvinar that project to the amygdala in macaques. Using AChE histochemical staining and VGLUT2 immunohistochemistry we were reliably able to identify the borders of pulvinar subdivisions within macaque monkeys. Consistent with past reports (Gutierrez et al. 1995; Stepniewska and Kaas 1997; Rovo et al. 2012; Balaram et al. 2013), we found that parts of the macaque inferior pulvinar extend above the BrSC. Further, when employing these histological stains in the context of our anatomical tract-tracing study, we were able to definitively identify which pulvinar subdivisions give rise to projections to the amygdala. Following injection of retrograde tracers into the lateral and/or basal nuclei of the macaque amygdala, retrogradely labeled cells were found in the medial pulvinar as well as divisions of the inferior pulvinar, including PIp and PIcl. Scattered retrogradely labeled cells were also evident in the lateral pulvinar. Thus, despite the emphasis of prior reports on medial pulvinar origins of amygdala projections (Jones and Burton 1976; Norita and Kawamura 1980; Rafal et al. 2015; Inagaki et al. 2023), our findings indicate that neurons in at least three subdivisions of the pulvinar—the medial, inferior, and to a lesser extent lateral—give rise to projections to the amygdala.

### Relative contributions of pulvinar divisions to amygdala projections

We found that of the total number of pulvino-amygdala projecting cells in our samples, the highest proportion originated within the medial pulvinar, with the next highest proportion within the PIp subdivision of the inferior pulvinar. Far fewer cells originated from PIcm, PIcl, or PL (Table 2).These findings should be interpreted with caution for at least three reasons. First, as noted in the Methods section, the study was not designed to be quantitative. Second, the two main pulvinar nuclei with amygdala projecting cells include both the largest (PM) and smallest (PIp) pulvinar nuclei, each with their own cell packing densities (Balaram et al. 2013). Third, the number of projecting cells may not relate to the magnitude of influence of the respective projections on amygdala function. To inform the relative impact of the medial and inferior pulvinar on the amygdala, future studies might assess the size of the projecting boutons and their location on the target cells, as well as identify the types of the cells in the amygdala that receive pulvinar inputs. These questions, however, are beyond the scope of our study.

### Comparisons with past studies in macaque monkeys

The general location of retrogradely labeled cells we observed in the pulvinar after placement of retrograde tracers in the amygdala was consistent with past reports (Jones and Burton 1976; Norita and Kawamura 1980; Rafal et al. 2015; Elorette et al. 2018; Inagaki et al. 2023). The major difference between the current study and prior reports is the revelation that the location of some of the labeled cells previously attributed to the medial pulvinar are actually within the inferior pulvinar. Further, we were able to identify specific subdivisions of the inferior pulvinar that project to the amygdala. This is a significant contribution as the functional and connectional features associated with the medial and inferior pulvinar are distinct and, therefore, the information that is transmitted through the medial versus inferior pulvinar is likely also distinct.

In brief, the medial pulvinar is considered to be a multimodal division of the pulvinar; in both New and Old World Monkeys, it shares connections with posterior parietal, temporal, and frontal cortex regions that include higher order visual, auditory, and somatosensory fields (Baleydier and Morel 1992; Romanski et al. 1997; Córdoba-Claros et al. 2025; for reviews see Homman-Ludiye et al. 2019; and Baldwin and Wise 2023). Further, cells in the medial pulvinar respond to multisensory stimuli (Mathers and Rapisardi 1973; Gattass et al. 1978; Vittek et al. 2023). The medial pulvinar also receives input from the intermediate layers of the SC (Benevento and Standage 1983), which contain cells with auditory and somatosensory receptive fields (Wallace and Stein 1996; Wallace et al. 1996) and receive inputs from subcortical auditory and somatosensory structures (see May 2006 for review). In primates, the intermediate layers of the SC also receive inputs from higher order visual, auditory and somatosensory cortical regions (Fries 1984; Lock et al. 2003; Wu et al. 2005; Collins et al. 2005; Cerkevich et al. 2014). The inferior pulvinar, on the other hand, is more aligned with visual sensory functions; it shares connections with both early and higher order visual cortical fields, and also receives input from superficial layers of the SC, which in turn are known to receive direct retinal input (for reviews see Kaas and Lyon 2007; Baldwin et al. 2017; Baldwin and Bourne 2017; Kaas and Baldwin 2019). Thus, a “fast” pathway for visual input to the amygdala seems more likely to arise from the inferior pulvinar than the medial pulvinar (but see below discussion). Physiological recordings from pulvinar subdivisions combined with proper identification of pulvinar recording locations would provide clarity on this issue.

### Comparison with other species

Tree shrews are the most studied of the extant close relatives of primates. Because of their phylogenetic position, they may help elucidate how the brains of rodents relate to the brains of primates (Fig. 1) (also see Baldwin and Petry 2025 for review). Tree shrews (Day-Brown et al. 2010) and rodents (Wei et al. 2015; Doron and LeDoux 1999) both possess pulvinar projections to the amygdala. The projections arise from pulvinar subdivisions identified as Pd (tree shrews) and LPcm (rodents). Based on shared connection patterns and chemoarchitectonic features (see Baldwin et al. 2017; Zhou et al. 2017; Baldwin and Bourne 2017; Kaas and Baldwin 2019), the Pd and LPcm of tree shrews and rodents are likely homologous to PIp and PIcm of macaques. Thus, our results reveal a retained and likely homologous projection from the visual inferior pulvinar to the amygdala across rodents, tree shrews, and primates. In all species studied, the inferior pulvinar or its presumed homologues is also associated with visual response properties and input from the retinal-recipient, superficial layers of the SC.

We also found projections to the amygdala from divisions of the pulvinar that do not have clear homologues in nonprimate species. This is especially clear with respect to the projections to the amygdala from the medial pulvinar, which is considered to be a primate specialization (Preuss 2007).

Finally, we found scattered amygdala-projecting neurons within another inferior pulvinar division, PIcl. Like the medial pulvinar, the PIcl does not have a clear or known homologue in other mammalian species outside the primate line. Though PIcl has historically been assigned to the inferior pulvinar, it could potentially be a part of the lateral pulvinar. The latter proposition is based on the punctate topographic terminations of SC projections to PIcl, similar to projections to the lateral pulvinar, and connections with ventral stream cortical areas (see Kaas and Lyon 2007 for review). Projections to the amygdala from the lateral LP in rodents (LPl) or the central pulvinar in tree shrews (Pc), both thought to be homologous to the lateral pulvinar in primates (see Fig. 1), have not been reported. Regardless of whether PIcl is part of the inferior or lateral pulvinar, based on our current knowledge, it seems likely that this projection to the amygdala is unique to primates.

Consistent with past observations in rodents (LeDoux et al. 1984; Le Doux et al. 1990; Romanski and LeDoux 1992 ; Doron and LeDoux 1999; Linke et al. 1999) and humans (Keifer et al. 2015; Rafal and Koller 2025), we found amygdala-projecting cells within the medial geniculate nucleus, suprageniculate nucleus and the limitans. These findings imply conserved subcortical projection pathways for auditory, or multisensory (i.e., Sg) input in addition to visual inputs. Interestingly, a study by Hackett and colleagues (1998) showed a matching topographic pattern of connections between the medial pulvinar of macaques with higher order auditory cortical structures. This result suggests that there is a close alignment of the medial aspect of the medial pulvinar with the auditory system. Given that this part of the medial pulvinar also receives inputs from neurons in the intermediate layers of the SC (Benevento and Standage 1983), which have auditory responses (Wallace et al. 1996), one might speculate that projections from the medial pulvinar, especially from its medial aspect, are processing auditory signals en route to the amygdala. If so, the medial pulvinar may provide an auditory or multisensory input to the amygdala not seen in rodents, one in addition to the subcortical auditory input from the medial geniculate nucleus (LeDoux et al. 1984; Le Doux et al., 1990; Romanski and LeDoux 1992; Doron and LeDoux 1999; Linke et al. 1999).

### The value of VGLUT2 staining and functional considerations

VGLUT2 is a valuable stain not only for identifying the boundaries of inferior pulvinar nuclei, but also because of its ability to reveal key details in the connections between the SC and pulvinar. Within a given cell, *VGLUT2* mRNA labeling is present within the cell body and VGLUT2 protein labeling is present within the cell’s respective terminals. Specific to the projection from the SC to pulvinar/LP complex, VGLUT2 mRNA is found within the layers of the SC that project to the pulvinar/LP complex (Balaram et al. 2013, 2015; Baldwin et al. 2017) and VGLUT2 protein is present within the terminals of SC cells projecting to the pulvinar (Masterson et al. 2009; Chomsung 2008; Wei et al. 2011). Further, boutons that contain VGLUT2 label within the pulvinar, and specifically within the inferior pulvinar, show large driver-like profiles (Rovo et al. 2012). Therefore, it would seem reasonable that projections from the SC have the capacity to drive activity within the inferior pulvinar. Although the density of VGLUT2 boutons within PIp is robust (Balaram et al. 2013; Fig. 2), direct evidence of VGLUT2 + boutons contacting amygdala projecting cells within PIp has not been established.

### The unresolved pathway for fast processing of emotional stimuli

To date, findings derived from several separate studies have led to the proposed retino→tectal→pulvinar→amygdala route for the fast visual pathway. Though we provide evidence that neurons in specific divisions of the inferior visual pulvinar—regions known to receive input from the superficial SC—project to the amygdala, it remains unknown whether these cells are a relay point for fast visual information to reach the amygdala. Evidence that neurons in the pulvinar are active in response to images of faces and snakes (Le et al. 2013; 2014; Maior et al. 2010) and at a time scale far faster than responses within visual inferior temporal cortex (Maior et al. 2010) have not been tied to exact locations in the pulvinar (i.e., the inferior or medial pulvinar). That is, the reported locations of the electrodes within the pulvinar where fast neuronal responses were recorded were not assessed histologically in a manner that would distinguish the pulvinar subdivisions. Moreover, the physiological findings have not been linked to behavior. Indeed, none of the disparate findings surrounding the putative fast pathway have been linked in a compelling way. In addition to the lack of evidence for point-to-point linkage, discussed above, there are two enormous gaps in knowledge, the first relating to affective processing and the second to ‘fast’ processing: 1) the lack of a causal manipulation testing whether the inferior and/or medial pulvinar is essential for processing affective signals; and 2) the lack of a causal manipulation testing whether the inferior and/or medial pulvinar or amygdala is essential for providing enhanced speed of detection or enhanced speed of processing of affective signals.

Further, whether such a putative pulvino-amygdala “fast” pathway is the same as that which provides patients with V1 lesions the ability to perform affective judgements regarding objects and faces in their blind visual field is also unknown. Although pulvinar damage in patients with unilateral V1 damage has been reported to prevent affective blindsight (Bertini et al. 2018), and this finding goes some way towards linking the processing of affective signals to the pulvinar, this result needs confirmation from studies in animal models in which causal manipulations can be more precisely placed and documented.

Further, regarding the proposed retino→tectal→pulvinar→amygdala route for the fast visual pathway, many alternative pathways could either contribute to, or serve as, ‘the’ critical pathway. Several visual pathways to the amygdala that bypass the tectum (and involve the pulvinar) or bypass the pulvinar are possible. For example, there are direct retinal projections to the inferior pulvinar in primates. Though most of these projections terminate within PIm (where only two amygdala-projecting cells across all cases were observed in our study), some retinal projections do terminate within PIp (Itaya and Hoesen 1982; Nakagawa and Tanaka 1984; Warner et al. 2010; 2015). Thus, there is the possibility that retinal ganglion cells could provide a source of visual input to PIp (bypassing the SC) that is then relayed to the amygdala. In addition, the limitans in macaques receives projections from superficial layers of the SC (Benevento and Fallon 1975) and projects to the amygdala (Fig. 3 of present study). Thus, this route could bypass the pulvinar to supply visual information to the amygdala.

It remains an open question whether cortical or retinal inputs (or both) are driving the cellular activity of pulvinar projecting neurons within the superficial layers of the SC. Recent work by Yu et al. (2024) in awake macaques suggests that the cortical input to the SC is essential for short-latency neuronal responses to visual stimuli. Specifically, Yu and colleagues found that reversible inactivation of the lateral geniculate nucleus abolished visual responses within the SC. This contrasts somewhat to earlier studies in anesthetized monkeys where neuronal responses within the superficial layers of the SC, specifically within the layer that receives retinal input, were maintained after cooling or ablation of V1 (Schiller et al. 1974). There are no direct projections from the LGN to the SC; therefore, the abolished activity reported by Yu et al. 2024 within the SC is likely due to the downstream effects of LGN inactivity propagating to V1, or via the small set of geniculate projections to other extrastriate visual areas, and then back to the SC. Reversible inactivations, however, can yield effects on downstream regions that differ from those of permanent lesions (Otchy et al. 2015). Whether the dynamic described by Yu and colleagues (2024) undergoes significant changes after long standing lesions of V1 is not currently known for macaques. In the context of permanent lesions that give rise to blindsight, circuits involving V1, the SC and its projections to the pulvinar may have time to undergo recalibration of their neural dynamics to allow visual processing in the SC to proceed. Further, extrastriate projections to the pulvinar may also undergo plasticity. Both of these scenarios could result in an enhancement of the pulvinar’s influence on visual processing in the amygdala. Consistent with this idea, a human blindsight patient capable of responding to emotional stimuli showed significant changes to the white matter pathways leading from the pulvinar to the amygdala (Tamietto et al. 2012).

### Summary

In summary, we report a conserved projection between the inferior pulvinar nucleus of the thalamus and the amygdala of macaques, one homologous to that observed in tree shrews and rodents. In addition, as has been reported previously, we found projections from the medial pulvinar to the amygdala. Further experiments that directly, causally manipulate activity of cells in the different divisions of the pulvinar are needed to determine whether the conserved pathway is essential for providing visual information responsible for fast neuronal responses to emotional stimuli in the amygdala, or for providing visual information for patients that experience affective blindsight.

## Data Availability

Data supporting the findings are presented in the figures and tables of the manuscript.
