# Correction: A conserved pulvinar projection to the amygdala revealed in macaque monkeys (*Macaca mulatta*)

**DOI:** 10.1007/s00429-026-03181-x

**Published:** 2026-08-25

**Authors:** Mary K. L. Baldwin, Arya Mohanty, Alexander C. Cummins, Elisabeth A. Murray

**Affiliations:** Laboratory of Neuropsychology, National Institute of Mental Health, National Institutes of Health, Building 49, Suite 1B80, 49 Convent Drive, Bethesda, MD 20892-4415 USA

**Correction: Brain Structure and Function (2026) 231:93**

**https://doi.org/10.1007/s00429-026-03145-1**

In this article, the figure 3 has been incorrectly processed, for the completeness and transparency, the incorrect and correct figures have been given below.

Incorrect figure 3:

**Figure:**
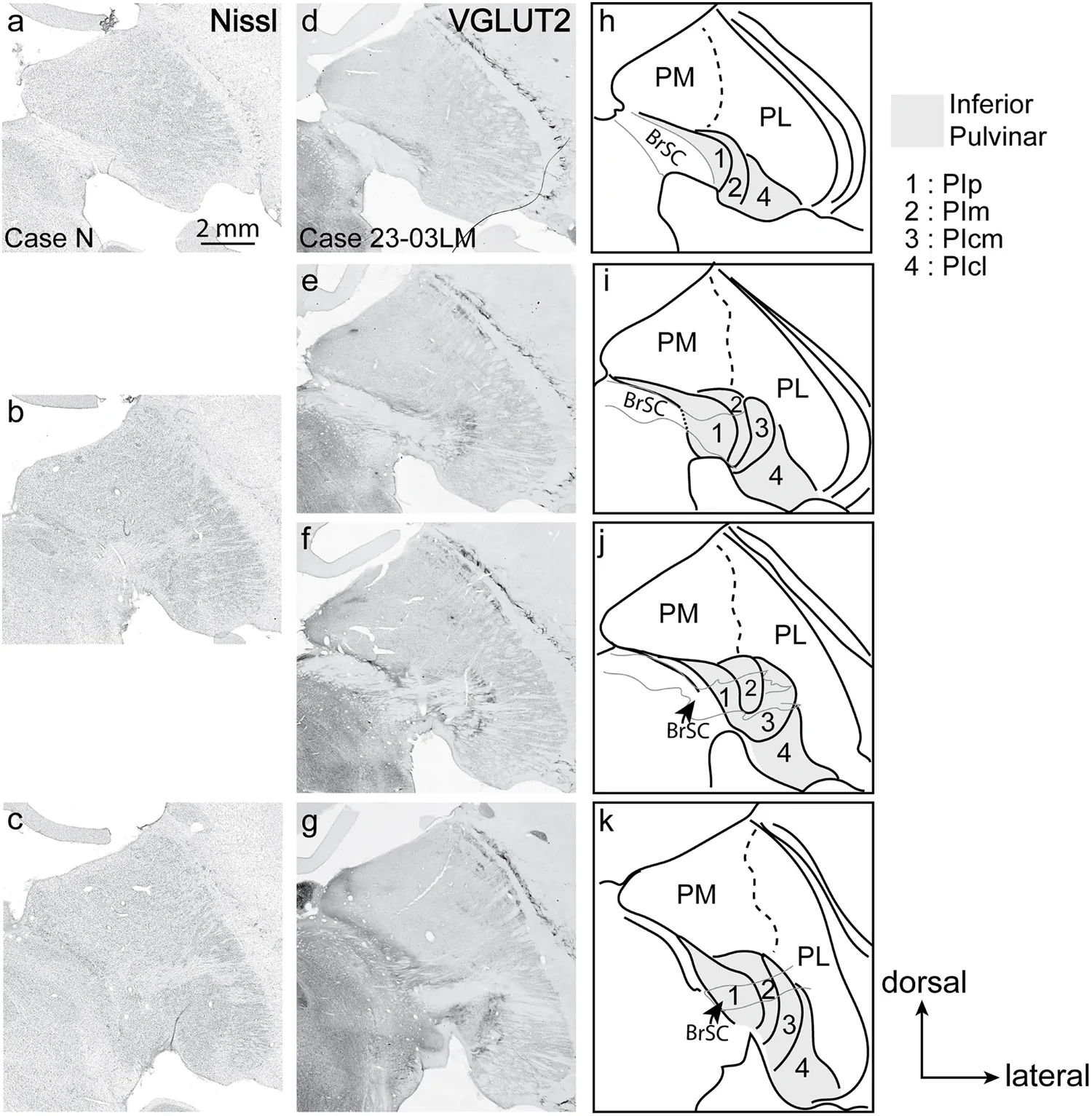

Correct figure 3:

**Figure:**
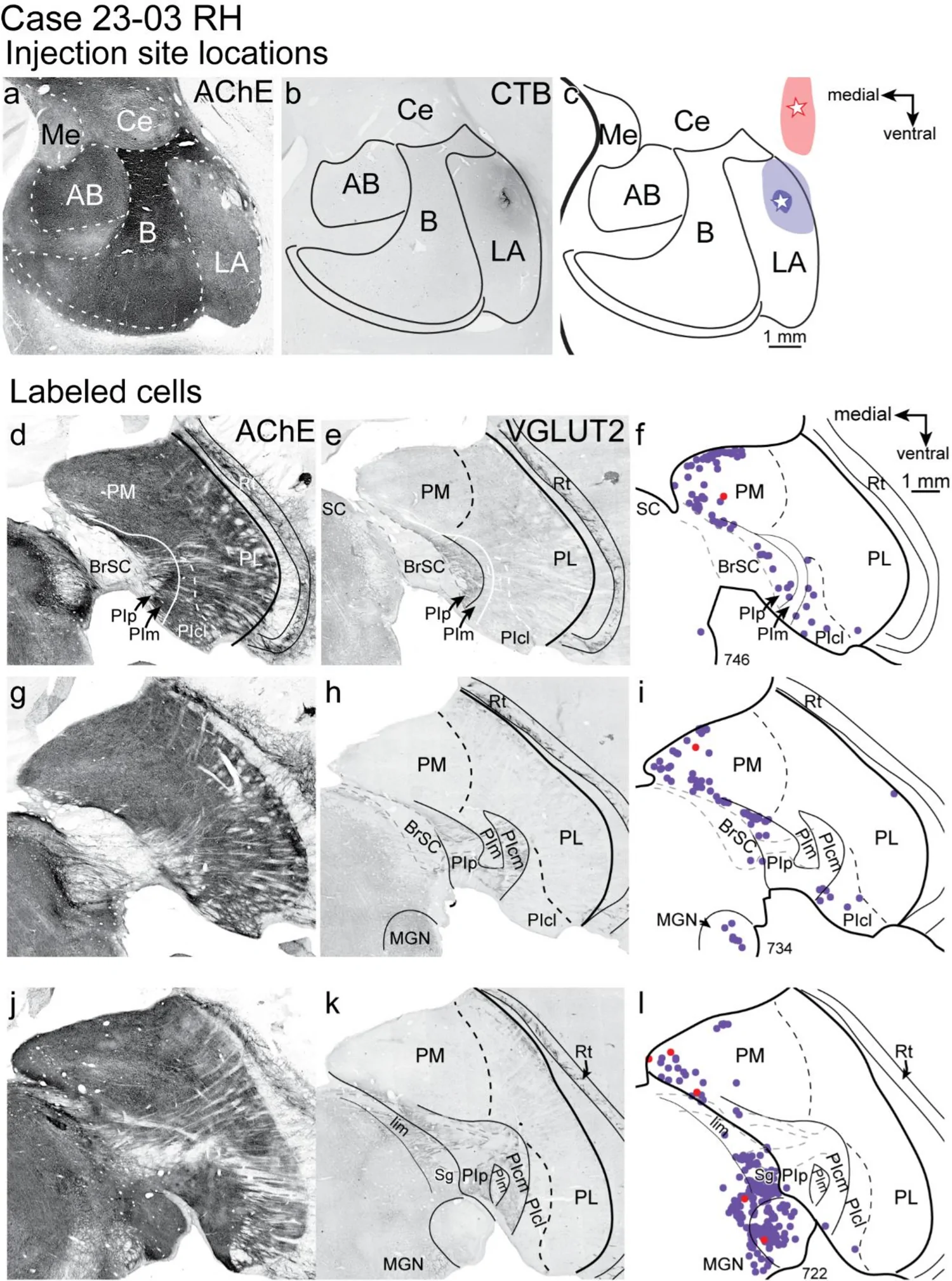

In this article, the reference “Romanski LM, LeDoux JE (1992) ……” should be appear before the “Kragel et al (2022)…”.

In this article, the reference “Keifer OP, Gutman D, Hecht E, Keilholz S, Ressler KJ (2015) ……” should be appear before the “Rosene et al (1986)…”.

The original article has been corrected.

